# Epidemiology of strongyloidiasis determined by parasite-specific IgG detections by enzyme-linked immunosorbent assay on urine samples using *Strongyloides stercoralis*, S. *ratti* and recombinant protein (NIE) as antigens in Northeast Thailand

**DOI:** 10.1371/journal.pone.0284305

**Published:** 2023-04-12

**Authors:** Chatanun Eamudomkarn, Sirowan Ruantip, Jiraporn Sithithaworn, Anchalee Techasen, Kulthida Y. Kopoolrat, Chanika Worasith, Phattharaphon Wongphutorn, Jeffrey M. Bethony, Thewarach Laha, Paiboon Sithithaworn

**Affiliations:** 1 Department of Parasitology, Faculty of Medicine, Khon Kaen University, Khon Kaen, Thailand; 2 Cholangiocarcinoma Research Institute (CARI), Khon Kaen University, Khon Kaen, Thailand; 3 Biomedical Science Program, Graduate School, Khon Kaen University, Khon Kaen, Thailand; 4 Wireless and Intelligent System for Dual-Use Applications Research Division (WISRD), National Science and Technology Development Agency (NSTDA), Pathum Thani, Thailand; 5 Faculty of Associate Medical Sciences, Khon Kaen University, Khon Kaen, Thailand; 6 Faculty of Public Health, Kasetsart University Chalermphrakiat Sakon Nakhon Province Campus, Sakon Nakhon, Thailand; 7 Department of Microbiology, Immunology & Tropical Medicine, George Washington University, Washington, DC, United States of America; Dokkyo Medical University, JAPAN

## Abstract

Detection of anti-*Strongyloides* IgG in urine by enzyme-linked immunosorbent assay (ELISA) for diagnosis of strongyloidiasis reportedly has comparable performance to conventional serum assays. Initial comparisons of urine assays using commercial ELISA kits designated for serology have shown its diagnostic potential but sub-optimal accuracy. In the present study, we optimized urine ELISA protocols based on different antigen types and evaluated their accuracies in determining the epidemiology of strongyloidiasis in Northeast Thailand. Paired urine and fecal samples of 966 individuals from the study community were collected for three consecutive days and tested for strongyloidiasis. We compared three ELISA protocols using different antigens including crude *S*. *stercoralis* antigen (Ss-ELISA), crude *S*. *ratti* antigen (Sr-ELISA) and recombinant NIE antigen (NIE-ELISA) and fecal examination by agar plate-culture (APCT) technique and formalin-ethyl acetate concentration technique (FECT). The optimized ELISA protocols using three different antigen sources yielded significantly higher prevalence rates of strongyloidiasis (58.9–65.1%) than those by fecal examination methods (19.7%). The prevalence of strongyloidiasis determined by ELISA protocols significantly increased with age (p value < 0.0001) and males had higher prevalence than females (p value < 0.0001). Diagnostic agreements between ELISA protocols were moderate (κ = 0.461–0.586) and the agreement between each ELISA protocol and fecal examinations were slight (κ = 0.139–0.210). The results obtained by urine ELISA protocols using three different antigens showed comparable diagnostic performances, provided further supports for the utility of urine as an alternative clinical specimen for diagnosis of strongyloidiasis.

## Introduction

Strongyloidiasis is a neglected tropical disease (NTDs), with transmission occurring globally in tropical and subtropical regions [1, 2]. It occurs widely in several countries of Southeast Asia particularly Thailand, Cambodia, Vietnam, and Malaysia [3, 4].

As a soil transmitted helminth, *Strongyloides stercoralis* invades the host through the skin and travels by various pathways to the small intestine of the host to reach patent infection. One unique characteristic of *S*. *stercoralis* is its autoinfection cycle whereby the parasite re-enters the enteral circulation or through the perianal skin without shedding larvae into the external environment [5, 6]. Larval output of *S*. *stercoralis* in human stool tends to be intermittent, which leads to conventional parasitological method having low sensitivity to detect chronic asymptomatic infection [6, 7]. Examination of multiple stool samples over several days is needed to improve the detection rate using specific methods such as the Baermann or Koga agar plate culture methods [8, 9]. As supplements to fecal examination, conventional as well as real-time polymerase chain reaction (PCR) tests can be used to detect parasite-specific DNA in serum, feces, and in urine with varying degrees of accuracies [10, 11].

Several serological tests have been reported that have increased diagnostic accuracy for strongyloidiasis [12, 13]. These methods utilized serum or plasma in indirect enzyme-linked immunosorbent assays (ELISA), usually employing crude extract of *S*. *stercoralis* third stage larvae as a homologous antigen [14, 15], or those from *S*. *ratti* or *S*. *venezuelensis* as heterologous antigens [16, 17]. ELISA tests using *S*. *ratti* or *S*. *stercoralis* larval antigens are comparable in their accuracy for serodiagnosis of strongyloidiasis in endemic area in northeast Thailand [18]. Recombinant *S*. *stercoralis* antigens, particularly a 31-kDa recombinant antigen (NIE) which was derived from a *S*. *stercoralis* third stage larva cDNA library [19], have also been used in ELISA to detect anti-*Strongyloides* IgG in serum [12, 20, 21].

Generally, the serological methods provide greater diagnostic accuracies than coprological methods, serving as the preferred detection methods for individuals at increased risk of lethal complications from chronic strongyloidiasis and patients awaiting transplantation or in immune therapy [2, 6]. However, serum-based diagnostic methods required venipuncture which is invasive and may be undesirable. Therefore, an alternative clinical sample which is easy to obtain is needed for sensitive diagnosis of strongyloidiasis for residents in *S*. *stercoralis* endemic areas.

In a search of clinical specimens other than blood, a urine-based ELISA was shown to have similar diagnostic performance to serum IgG for immunodiagnosis of strongyloidiasis [22]. The application of urine ELISA, using *S*. *ratti* as antigen in a nation-wide mapping of strongyloidiasis in Cambodia, was recently reported [23]. An assessment of different antigens in urine ELISA for diagnosis of strongyloidiasis suggested that commercial kits using *S*. *stercoralis* crude and recombinant protein (NIE) antigens had lower diagnostic performance than ELISA using *S*. *ratti* antigen [24]. However, these commercial ELISA kits were designed for serological tests (serum-based testing), therefore a specific and optimized protocol for urine analysis is needed. We hypothesize that fully optimized urine ELISA protocols for use of crude *Strongyloides* antigen as well as recombinant NIE antigens from *S*. *stercoralis* should have similar performances in diagnosis of strongyloidiasis.

In this study, we examined the community-based epidemiological patterns of strongyloidiasis regarding effects of age and sex of the local residents based on parasitological and immunological diagnoses in northeast Thailand. We also assessed and compared the detection rate and agreement of ELISA protocols using three different antigen sources i.e. the *Strongyloides* NIE recombinant antigen (NIE-ELISA), crude *S*. *stercoralis* (Ss-ELISA) and crude *S*. *ratti* antigen (Sr-ELISA). The prevalence of strongyloidiasis over three consecutive days in previous study showed that the variation of prevalence by urine ELISA was low while there were significant variations by parasitological examination [25]. Comparisons of the ELISA protocols based on different antigen types should reveal the utility and reliability of urine ELISA in clinical diagnosis as well as for population screening of strongyloidiasis.

## Materials and methods

### Ethical statement

The study protocol concerning the participant enrollments as well as clinical sample collection and examination were approved by the Ethics Committee of Khon Kaen University, Khon Kaen, Thailand (HE654013) in compliance with the Declaration of Helsinki. The project activity was explained and written informed consent was obtained from each participant. The study included residents of all sexes aged > 18 years. All parasite-infected participants discovered during the project operation were given specific anthelmintic treatments. All sample analyses were anonymized, and blinding was applied during sample analysis and result interpretations.

The protocol for laboratory animal experiments required for production of *S*. *ratti* parasite antigen was approved by the Institutional Animal Ethical Committee, Khon Kaen University (IACUC-KKU-100/61). The experiment animals were handled in accordance with good animal practice according to the guidelines for the Care and Use of Laboratory Animals of the National Research Council of Thailand.

### Sample population and clinical sample collection

The sample population participating in this study resided in Nong Krung Sri District (NKS), Kalasin Province, Northeast Thailand. The study area in this study was in nearby sub districts (Tambon) in NKS reported previously [25]. This study is a prospective cross-sectional study conducted in 2018 to measure uro-and copro-prevalence of strongyloidiasis for 3 consecutive days. Individuals with clinical background of chronic diseases, current steroid treatment, kidney disease, and cancers were excluded. The personal history and demographic data were obtained during the interview. Therefore, individuals with prior *Strongyloides* sp. infection were excluded from the study. The original invitations for enrollment included 1,025 people and 966 individuals enrolled, consisted of 463 were men and 503 were women with a median age of 53 (range 18–86). Each study participant was requested to provide paired specimens of feces and urine samples for three consecutive days. Urine samples were collected in the morning and were transferred into sample tubes containing Sodium azide as preservative (0.1%) and kept in insulated ice box. Fecal samples were collected in wide-mouthed containers and kept in an insulated box at ambient temperature. Any vehicle or person having contact with the sample box and accessories was cleaned with 1% Lugol’s iodine or 70% isopropyl alcohol solution to prevent transmission of pathogens. Within the same day, urine and fecal samples were transported to the laboratory at Khon Kaen University for processing.

Positive and negative control sera of strongyloidiasis kept in the Department Biobank were collected from our previous study [22]. Negative control sera were obtained from individuals in non-endemic area, central part of Thailand and were negative by repeated FECT and APCT.

### Fecal examination methods

Fecal samples were examined for parasitic infections using both the agar plate-culture technique (APCT) and the formalin-ethyl acetate concentration technique (FECT). The procedure for APCT was as described elsewhere [26]. Briefly, approximately 4 g of fecal sample was placed on 1.5% nutrient agar in a plastic petri dish and incubated at 25°C for 2–4 days. The culture dishes were sealed with adhesive tape which prevents larvae from crawling out and provides safe conditions for a technician. The presence of worms was assessed by washing the surface of each plate with 10% formalin, transferring the wash into a test tube then centrifuging and examining the sediment as a wet preparation under a light microscope.

For FECT, the method was described previously [27]. Briefly, approximately 2 g of feces already fixed with 10% formalin was processed for examination. The procedure began with a thorough mixing of fecal material into a suspension which is then strained through gauze into a test tube. To this was added 3 mL of ethyl acetate to extract fat from feces. The test tube was then vigorously shaken. After centrifugation for 5 minutes, the supernatant was discarded, and the sediment was re-suspended in 10% formalin. The final fecal suspension was examined for the presence of parasites in triplicate examinations.

### Antigen preparation

Preparation of crude *S*. *ratti* and *S*. *stercoralis* antigens was performed as previously described [15, 18]. For *S*. *ratti*, feces of Wistar rats previously infected with *S*. *ratti* were cultured to produce third-stage filariform larvae using a filter-paper culture method [28]. In the case of *S*. *stercoralis*, fecal samples from *S*. *stercoralis*-positive subjects in endemic area from our previous study [18] were cultured on APCT plates and larvae subsequently harvested. The third stage larvae of *S*. *stercoralis* and *S*. *ratti* were separately pooled and stored as sediments at -20°C until required for antigen preparation.

The larval sediment (*S*. *ratti* or *S*. *stercoralis*) was thawed and suspended in phosphate-buffered saline (PBS) pH 7.4 containing protease inhibitor mix (GE Healthcare, Bio-Sciences Corp., USA) and then disrupted by sonication. The larval homogenate was allowed to further solubilize at 4°C overnight and then was centrifuged at 15,000 rpm for 30 minutes at 4°C. The protein concentration of the supernatant was measured using the Bradford protein assay (Bio-Rad Laboratories Inc., CA), the solution aliquoted and stored at −20°C until used as crude *S*. *ratti* and *S*. *stercoralis* antigens in the ELISA. The NIE recombinant protein prepared as described elsewhere [19] was kindly provided by T. Nutman, NIAID, NIH, Bethesda, MD.

### Enzyme-linked immunosorbent assay

The protocols for urine ELISA using *S*. *ratti* (Sr-ELISA) or *S*. *stercoralis* (Ss-ELISA) crude antigens were similar to those in our previous report [22, 24]. In case of the *S*. *stercoralis* NIE recombinant protein, the protocol (NIE-ELISA) used in this trial were modified from previous study [21]. For optimization of NIE-ELISA, chessboard titrations for antigen concentration, serum dilution and conjugate dilution were carried out by determining the lowest signal-to-noise ratio between defined strong *S*. *stercoralis*-positive and normal human urine samples. The optimal conditions were subsequently used for sample analyses.

The ELISA procedure began by coating the plates with *S*. *ratti* antigen (5 μg/mL), *S*. *stercoralis* antigen (1 μg/mL) or NIE antigen (0.3 μg/mL) at 4°C overnight. Ninety-six-well microtiter plates (Maxisorp; Nunc, Roskilde, Denmark) were used. The plates were washed with washing buffer (PBS pH 7.2, containing 0.05% Tween-20) twice before blocking by 3% skimmed milk (Invitrogen, CA, USA) and 0.5% Tween-20 in PBS for about 2 hours at ambient temperature. Undiluted urine samples (100 μL/well) were added in duplicate and incubated 1 hour at 37°C. A triplicate washing was performed and then 100 μL/well of 1:4,000 dilution of horseradish peroxidase conjugate goat anti-human IgG (Abcam Inc., UK) was added. After 1 hour at 37°C, the plates were washed 3 times with washing buffer. Subsequently, 100 μL/well of substrate solution was added and the plates incubated (Sr-ELISA and Ss-ELISA) for 1 hour at room temperature in a humidified dark box using *o*-phenylenediamine solution (Sigma, St. Louis, MO, USA). In the case of NIE-ELISA, 100 μL/well of 3,3′,5,5′-tetramethylbenzidine solution (Sigma, St. Louis, MO, USA) was used and the plates incubated for 30 minutes. The final step was to stop the reaction by adding 4N sulfuric acid (50 μL/well), then to measure the optical density (OD) of each well using an ELISA reader (TECAN Sunrise, Austria) at 492 nm for Sr-ELISA and Ss-ELISA and 450/620 nm for NIE-ELISA.

In order to improve the accuracy of ELISA, the OD values were used to calculate the arbitrary antibody unit in the ELISA protocol reported previously [22, 29, 30]. Antibody levels of urine were calculated from OD and expressed as units based on a standard curve made from three-fold serially diluted pools of high-titer positive sera from strongyloidiasis patients. To construct the standard curve, pooled sera of proven cases of strongyloidiasis, determined by serum ELISA and larval positive assessed by APCT or FECT, were three-fold serially diluted (1:3,000 to 1:2,187,000) in duplicates with included in the ELISA plate. As antibody units, a value of 7,290 U was arbitrarily assigned to the 1:3,000 dilution, a value of 10 U to the 2,187,000 dilution. The relationship between OD values and antibody units (unit/ml) were incorporated into the regression equation for estimation of antibody unit. These pooled positive sera were used as internal controls with negative serum controls and blanks for each microtiter plate. Analysis of receiver operating characteristic curves was used to determine the cut-off value by using urine samples from 30 strongyloidiasis cases and 30 uninfected individuals (determined by APCT and FECT). The cut-off values for Sr-ELISA (101 units/mL), Ss-ELISA (283 units/mL) and NIE-ELISA (428 units/mL) were used to determine the negative and positive diagnostic properties. The cross-reactions of parasitic infections other than *S*. *stercoralis* were assessed to determine the specificity of the ELISA tests. In addition to the previous reports for Ss-ELISA and Sr-ELISA [18, 22], a separated group of 31 subjects were used to assess the cross reactivity of NIE-ELISA. These subjects were screened for parasitic infection by fecal examination (APCT and FECT). These included individuals infected with *O*. *viverrini* (n = 21), *Taenia* spp. (n = 4), *Trichuris trichiura* (n = 1), *Echinostoma* sp. (n = 3), Minute intestinal fluke (n = 1), and hookworms (n = 1).

### Statistical analysis

The data generated in this study were entered into an Excel spread sheet and analyzed using SPSS version 21.0 software (IBM Corporation, Armonk, NY). Data on prevalence of strongyloidiasis obtained from daily as well as cumulative test results were compared using McNemar’s chi-square tests. Chi-square tests were also used to assess the association of prevalence with age and sex of the study participants. The diagnostic performance of each ELISA protocol was calculated as detection rate. Diagnostic agreements between diagnostic tests were determined based on Cohen’s kappa value (κ) that is interpreted as follows: almost perfect agreement (0.81–1.00), substantial (0.61–0.80), moderate (0.41–0.60), fair (0.21–0.40), slight (0.00–0.20), and poor agreement (< 0.00) [31]. The correlation between IgG antibody levels in serum and urine was assessed using the regression coefficient of the linear regression model. The differences of level of anti-*Strongyloides* IgG between in individuals with and without co-infecting *O*. *viverrini* were assessed by Mann–Whitney U test and chi-square tests. MedCalc version 11.6.1.0 software (Ostend, Belgium) was used to calculate the cut-off values based on ROC curves to obtain the area under the curve (AUC) value for each ELISA protocol. The cut-off values for Sr-ELISA (101 units/mL), Ss-ELISA (283 units/mL) and NIE-ELISA (428 units/mL) were used to determine the negative and positive diagnostic results. Analytical graphs were constructed using GraphPad Prism version 7.0 (Graph Pad Software Inc, San Diego, CA, USA). All statistical tests were considered significant at p values less than 0.05.

## Results

### Prevalence of parasitic infections determined by fecal examinations and urine ELISA assays

Prevalence rates of parasitic infection determined by fecal examinations (APCT and FECT) in 966 individuals were as follows: *S*. *stercoralis* (19.7%), *O*. *viverrini* (12.6%), hookworms (2.6%), *Blastocystis hominis* (0.4%) and *Giardia duodenalis* (0.4%).

**Fig 1:**
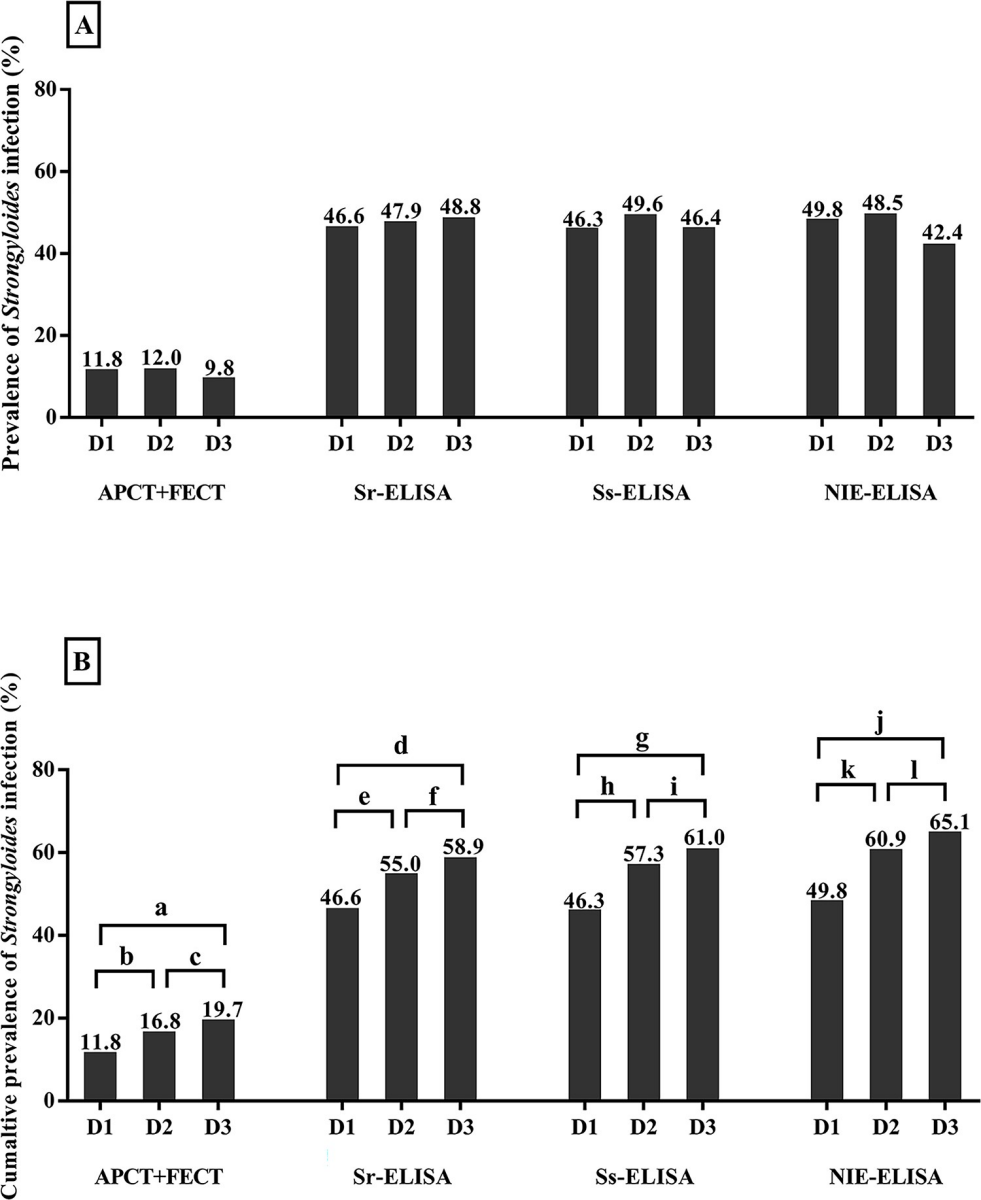
Daily and cumulative prevalence rates of *Strongyloides* infection according to fecal examination and urine ELISA methods (n = 966). Panel A shows the prevalence rates for each of three successive days (D1: day1, D2: day2, D3: day3). Panel B shows the cumulative prevalence rate for three days (D1: day1, D2: day 1 and 2, D3: day 1, 2 to day 3). The small letter symbols (a-l) represent the McNemar’s chi-square value for a statistical comparison between pairs of prevalence estimates with p value < 0.0001; a = 66, b = 44, c = 28, d = 106, e = 38, f = 77, g = 130, h = 101, i = 35, j = 151, k = 109, and l = 47.

For *S*. *stercoralis*, daily and cumulative prevalence rates over three consecutive days determined by fecal examinations and urine ELISA are compared in Fig 1. The daily prevalence rates according to fecal examinations varied slightly from 9.8 to 12.0% over three consecutive days, but differences were not statistically significant (p value > 0.05, Fig 1A). Urine ELISA tests based on three different antigens had comparable daily prevalence rates with the rates approximately 4–5 times higher than those by fecal examinations.

The cumulative prevalence of *S*. *stercoralis* infection according to fecal examinations increased significantly between examinations over three consecutive days from 11.8% to 19.7% (McNemar test = 28–66, p value < 0.0001, Fig 1B). By urine ELISA, the prevalence rates also significantly increased over time in all three assay protocols (Sr-ELISA, Ss-ELISA and NIE-ELISA). All urine ELISA protocols indicated far higher prevalence than did fecal examinations (McNemar test = 336–354, p value < 0.0001). There was no difference in prevalence rates between different protocols of urine ELISA (p value > 0.05).

### Associations between prevalence of strongyloidiasis with age and gender

**Fig 2:**
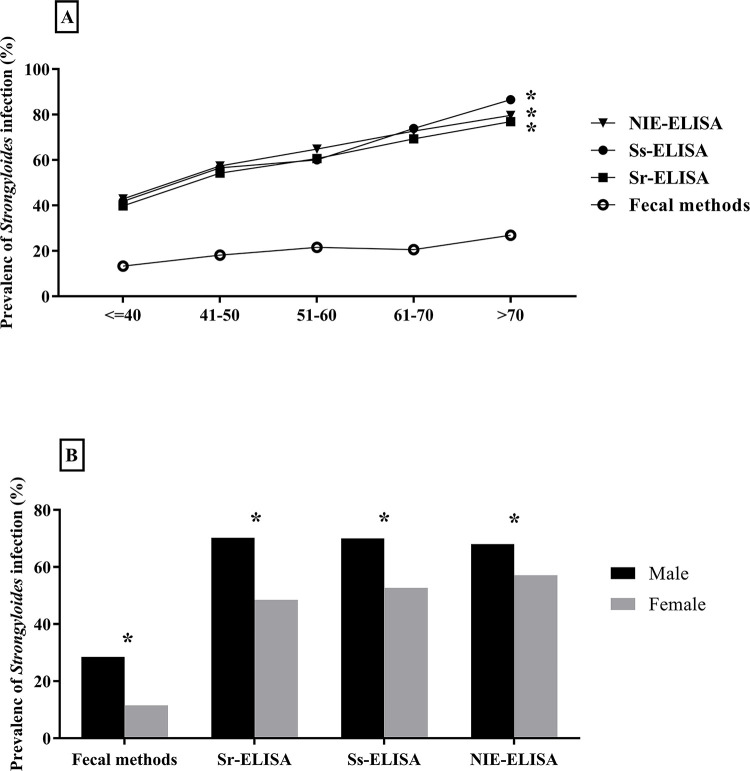
Prevalence profiles of *S*. *stercoralis* infection determined by different diagnostic methods arranged by age (Panel A) and gender (Panel B). * Stands for p value < 0.0001 obtained by chi-square test.

The prevalence profiles of strongyloidiasis arranged by age and gender are presented in Fig 2A and 2B, respectively. All the urine ELISA protocols showed significantly higher prevalence in male than female (chi-square test = 30.3–46.8, p value < 0.0001) and increasing with age of the participants (chi-square test = 32.7–44.5, p value < 0.0001). Fecal examination also showed that prevalence of strongyloidiasis was higher in males (chi-square test 43.988, p value < 0.0001) but no significant relationship between prevalence of strongyloidiasis and age was found (chi-square = 5.6, p value = 0.2).

### Detection rates of urine ELISA

Detection rates by the urine ELISA protocols based on antigens prepared from *S*. *ratti*, *S*. *stercoralis* and recombinant protein (NIE) of *S*. *stercoralis* for diagnosis of strongyloidiasis are shown in Table 1. Sr-ELISA had a higher detection rate than that by Ss-ELISA (McNemar’s chi-square test, p<0.05) and similar to that by NIE-ELISA.

**Table 1:** Detection rates of urine ELISA protocols based on antigens prepared from *S*. *ratti*, *S*. *stercoralis* and recombinant protein (NIE) of *S*. *stercoralis* evaluated against the fecal examination (n = 966). Data shown are detection rate and 95% confidence intervals.

Diagnostics	Detection rate (95% CI)
Sr-ELISA	88.4 (82.8–92.4)
Ss-ELISA	81.1^a^ (74.6–86.2)
NIE-ELISA	83.2 (76.9–88.0)

^a^ significant difference from Sr-ELISA (p<0.05) by McNemar’s chi-square test.

### Agreements between ELISA protocols and fecal examination

Comparisons between each pair of ELISA protocols yielded kappa values (κ = 0.461–0.586) in the range of moderate agreement (Table 2). The agreements were slight to fair (κ = 0.139–0.210) between the urine ELISA protocols and fecal examination. Quantitative comparisons between levels of parasite-specific IgG in urine measured by each pair of ELISA protocols showed significant correlations (S1 Table).

**Table 2:** Diagnostic agreements between urine ELISA protocols and fecal examination methods and between pairs of different antigen-based ELISA protocols. Data shown are Cohen’s kappa values (based on data from 966 individuals).

Diagnostic methods	κ coefficient	SE ^a^	95% CI ^b^	p value
Fecal methods & Sr-ELISA	Fair (κ 0.210)	0.021	0.169–0.250	<0.0001
Fecal methods & Ss-ELISA	Slight (κ 0.139)	0.021	0.096–0.173	<0.0001
Fecal methods & NIE-ELISA	Slight (κ 0.143)	0.020	0.096–0.171	<0.0001
Sr-ELISA & Ss-ELISA	Moderate (κ 0.586)	0.027	0.540–0.644	< 0.0001
Sr-ELISA & NIE-ELISA	Moderate (κ 0.481)	0.029	0.394–0.509	< 0.0001
Ss-ELISA & NIE-ELISA	Moderate (κ 0.461)	0.029	0.421–0.536	< 0.0001

^a^ SE stands for standard error

^b^ 95% CI stands for lower and upper 95% confidence interval

### Relationship between reactivity of urine ELISA and parasitic infection

**Fig 3:**
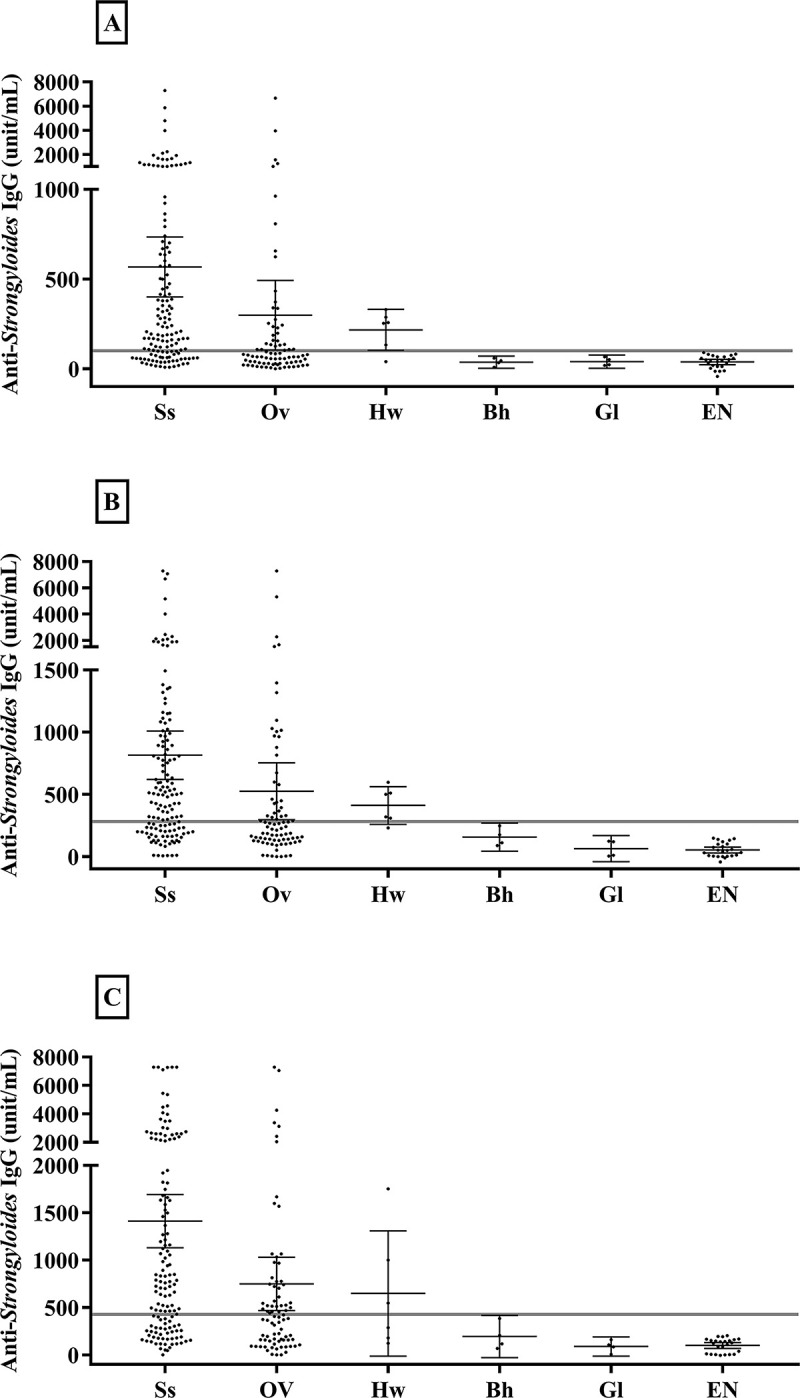
Distribution of anti-*Strongyloides* IgG levels among individuals with various parasitic infections determined by fecal examination. Urine analysis protocols by Sr-ELISA (A), Ss-ELISA (B), NIE-ELISA (C). Points shown (dots) are individual values of IgG antibody (units/mL of urine). The long horizontal lines indicate the cut-off values of IgG level. Labels for parasitic infections; Ss: *S*. *stercoralis*, Ov: *O*. *viverrini*, Hw: hookworms, Bh: *B*. *hominis*, Gl: *Giardia duodenalis*, and EN: endemic negative. Short horizontal lines indicate means with lower and upper 95% confidence intervals.

Fig 3 shows the distribution of anti-*Strongyloides* IgG levels among individuals with various parasitic infections determined by fecal examination. Of those individuals positive for *S*. *stercoralis* according to fecal examination, Sr-ELISA also identified 70.7% as positive. Corresponding values for Ss-ELISA and NIE-ELISA were 65.7% and 67.1%, respectively. Of those positive for *O*. *viverrini*, urine ELISAs yielded values above the cut-off for 46.9% (NIE-ELISA) and 39.5% (for both Sr-ELISA and Ss-ELISA). In individuals with hookworm infection, 3 of 6 cases (50%) yielded values above the cut-off in NIE-ELISA and 5 of 6 cases (83.30%) by Ss-ELISA or Sr-ELISA. No positive test was seen for *B*. *hominis* (n = 4) and *G*. *doudenalis* (n = 4) by any of the 3 ELISAs.

Since opisthorchiasis was the second most common infection identified in the study population, effects of co infection with *O*. *viverrini* on levels of anti-*Strongyloides* IgG were analyzed and results are shown in **S1 Fig**.

There were no significant differences for the level of anti-*Strongyloides* IgG determined by each ELISA protocol in individuals with and without opisthorchiasis (Mann–Whitney U test, p value > 0.05). The levels of anti-*Strongyloides* IgG in the participants with and without opisthorchiasis (mean ± standard error) by Sr-ELISA were 298.3 ± 97.4 unit/mL and 310.6 ± 33.9 unit/mL, by Ss-ELISA were 524.7 ± 114.5 unit/mL and 548.9 ± 40.4 unit/mL, and by NIE-ELISA were 749.7 ± 141.6 unit/mL and 772.7 ± 47.8 unit/mL.

For positive rates of anti-*Strongyloides* IgG, no differences were found between the participants with and without *O*. *viverrini* infection determined by fecal examination (chi-square test, p value > 0.05). In the participants with and without opisthorchiasis, the positive rates were 39.5% v.s.40.3% by Sr-ELISA, 39.5% v.s. 42.4% by Ss-ELISA and 43.2% v.s. 41.1% by NIE-ELISA.

### Cross reactivity of ELISA protocols to other parasitic infections

The reactivity of ELISA protocols to other parasitic infection based on previous publications [18, 22] and current paper is shown in Table 3. The cross reaction with *O*. *viverrini* were seen in most protocol in serum Ss-ELISA [18], urine Sr-ELISA [22] and urine NIE-ELISA. Reactivity was also seen in *Taenia* sp. and hookworms for NIE ELISA.

**Table 3:** Reactivity of serum and urine ELISA protocols based on *S*.*stercoralis*, *S*. *ratti* and *S*. *stercoralis* NIE antigen.

Parasite	*Strongyloides* antigen for ELISA		
	*S*. *stercoralis* ^a^	*S*. *ratti* ^b^	*S*. *stercoralis* NIE ^c^
*Opisthorchis viverrini*	5/20	2/15	3/21
*Taenia* spp.	0/2	0/7	2/4
*Trichuris trichiura*	-	0/4	0/1
*Echinostoma* sp.	-	0/6	0/3
Minute intestinal fluke	-	0/8	0/1
Hookworms	0/2	-	1/1
*Ascaris lumbricoides*	0/2	-	-

^a^ Eamudomkarn et al., 2015 (serum ELISA)

^b^ Eamudomkarn et al., 2018 (urine ELISA)

^c^ This study (urine ELISA)

## Discussion

The age-prevalence profiles of strongyloidiasis determined by detection of *Strongyloides* IgG in urine using three different preparations of *Strongyloides* antigen were similar. Urine ELISA yielded significantly higher prevalence rates of strongyloidiasis than those estimated by standard fecal examinations. Agreements between ELISA protocols in diagnosis for strongyloidiasis were moderate (κ = 0.461–0.586) while diagnostic agreements between each ELISA protocol and fecal examinations were slight (κ = 0.139–0.210). The Sr-ELISA and NIE-ELISA protocols had slightly better detection rate in diagnosis of strongyloidiasis than Ss-ELISA. The results that different *Strongyloides* antigen based ELISAs achieved similar diagnosis provided further support for the utility and reliability of urine for diagnosis of strongyloidiasis and future control program. In Thailand, currently no specific community-based control program targeting strongyloidiasis is being implemented. Specific laboratory diagnostic services for serodiagnosis i.e. parasitological method (APCT) and serodiagnosis are available in tertiary care health centers or provincial hospital settings.

The unit-based ELISA protocol similar to those reported previously from our group used in this study [22, 29, 30]. Antibody levels in urine was arbitrarily assigned as unit based on a standard curve and were calculated from the OD. The calculation of arbitrary ELISA units/milliliter for the positive samples by 4-parameter logistic (4-PL) regression analysis of the standard curve were reported in previous studies [32–34]. These studies demonstrated arbitrary ELISA method with high degree of accuracy, reduce the variability between assays and operators, therefore, providing a more precise measurement of antibody concentration for patient sera.

Previous comparisons between different *Strongyloides* antigens for serodiagnosis of strongyloidiasis in serum ELISA found that *S*. *stercoralis* and *S*. *ratti* antigens were more sensitive than the NIE antigen (in studies mostly from USA and Latin America) [12] or were comparable in sensitivity [20]. Previous reports from Southeast Asia, especially Thailand, showed varying diagnostic performances using antigen prepared from *S*. *stercoralis* [35, 36], *S*. *ratti* [22, 24] and an *S*. *stercoralis* recombinant protein [24, 37]. We have previously reported similar diagnostic performances of Sr-ELISA and Ss-ELISA in a rural community in Khon Kaen Province, Northeast Thailand [18].

In the case of urine analysis, Sr-ELISA was used in a mass population screening in Cambodia to assess risk factors and geographical distribution of strongyloidiasis [23]. Although crude antigens from *S*. *stercoralis* and the recombinant NIE antigen exhibit considerable sensitivity for serodiagnosis [12, 20, 21, 24, 38], they have not previously been evaluated in urine ELISA assays. In this study, crude antigens prepared from either *S*. *ratti*, *S*. *stercoralis* and the recombinant protein (NIE) of *S*. *stercoralis* showed comparable results. The levels of antigen-specific IgG in urine were significantly and quantitatively related across the three forms of antigen. Moreover, the ongoing study from our group showed significant reductions of *S*.*ratti*-specific IgG in urine at 3 months post treatment (unpublished data). The evaluation of antibody level in urine by these ELISA protocols required further study.

It is interesting to note that when using the standard reference fecal examination, the detection rates (88%) of Sr-ELISA were high in the present study. Previous studies in Northeast Thailand found a sensitivity of 93% and specificity of 41% [22] and sensitivity 83% and specificity 47% [24] of urine ELISA. In addition to possible variations in laboratory conditions for ELISA, the levels of endemicity in terms of prevalence of strongyloidiasis in the study areas i.e., 28%, 64.8% [22, 24] and 19.7% (this study) may influence the detection rate determined by ELISA.

Similar to previous reports in which urine ELISA as well as serum ELISA was used for diagnosis of strongyloidiasis, we found cross reactivity 3 ELISAs with opisthorchiasis and also hookworms. Since the study community is an endemic area for opisthorchiasis, observed positive ELISA for strongyloidiasis in parasitologically proven opisthorchiasis cases is not unexpected. Comparisons of urine IgG by three different *Strongyloides* antigens in the ELISA protocols showed no difference between co-infection with and without opisthorchiasis. The results in this study suggested that co-infection with *O*. *viverrini* has no effect on the *Strongyloides*-specific IgG in urine for diagnosis of strongyloidiasis. Since other intestinal or blood, and tissue nematodes i.e. *Trichuris*, and *Ascaris* and filariasis are currently not present in the study community, tests for cross reactions with these parasites have not been performed.

The association between age of the participants and prevalence of strongyloidiasis observed in this study is comparable with previous reports [23, 39–42]. The prevalence among males was greater than females, which is also similar to previous reports [40–43]. The gender or age-related pattern of infection may be related to the cumulative possibility of soil contact through time, such as among agriculturists, or because older individuals had long-term chronic infection in this endemic area.

There were several limitations we acknowledge in this study. First is the false negative diagnosis by urine ELISA in parasitological positive cases. Similar observations have been reported in previous serum-based diagnostic studies and were believed to be associated with low immunity [44] or excessive dilution of urine samples [45, 46]. Since urine composition is related to water intake, future work to concentrate urine samples prior to ELISA may help to alleviate the problem of false negative results [47]. Second, although analysis of urine and serum samples by ELISA had high concordance for qualitative and quantitative diagnosis of strongyloidiasis [22, 24, 47], this needs to be confirmed in different endemic settings. Third, additional confirmation other than parasitological, such as DNA detection by fecal PCR or immune-complex detection [48, 49] in cases of false-positive urine ELISA is needed to better evaluate the performance of parasite-specific IgG detection in serum as well as in urine. Last, more cross reactivity of the ELISA protocols to other parasites is needed.

In conclusion, the results in this study indicated that urine ELISA protocols were more sensitive than fecal examination. Concordance was observed between ELISA protocols using different sources of antigens for diagnosis of strongyloidiasis. The quantitative and qualitative diagnostic parameters by ELISA protocols using *S*. *stercoralis*, *S*. *ratti* and NIE antigen for ELISA were comparable. These results strengthen the utility of urine ELISA for diagnosis of strongyloidiasis.

## Supporting information

S1 Fig Distribution of anti-*Strongyloides* IgG by Sr-ELISA (A), Ss-ELISA (B), and NIE-ELISA (C) among individuals with and without parasitologically confirmed opisthorchiasis. The gray horizontal lines represent the cutoff values. Short black lines indicate the mean with lower and upper standard error. Ov- means negative *O*. *viverrini* egg; Ov+ means positive *O*. *viverrini* egg.
(TIF)

S1 Table Correlations between levels of parasite-specific IgG in urine determined by ELISA protocols using antigen prepared from *S*. *ratti* (Sr-ELISA), *S*. *stercoralis* (Ss-ELISA) and recombinant protein NIE antigen (NIE-ELISA). Data shown are correlation coefficients between antibody levels (IgG antibody unit/mL) from each pair of ELISA protocols according to Spearman correlation tests and p values (based on data from 966 individuals).
(DOCX)
