# Supplementary figures and images for: Epidemiology of strongyloidiasis determined by parasite-specific IgG detections by enzyme-linked immunosorbent assay on urine samples using Strongyloides stercoralis, S. ratti and recombinant protein (NIE) as antigens in Northeast Thailand
Source: PLoS One. 2023 Apr 12;18(4):e0284305. doi: 10.1371/journal.pone.0284305 (PMC10096234; doi:10.1371/journal.pone.0284305)

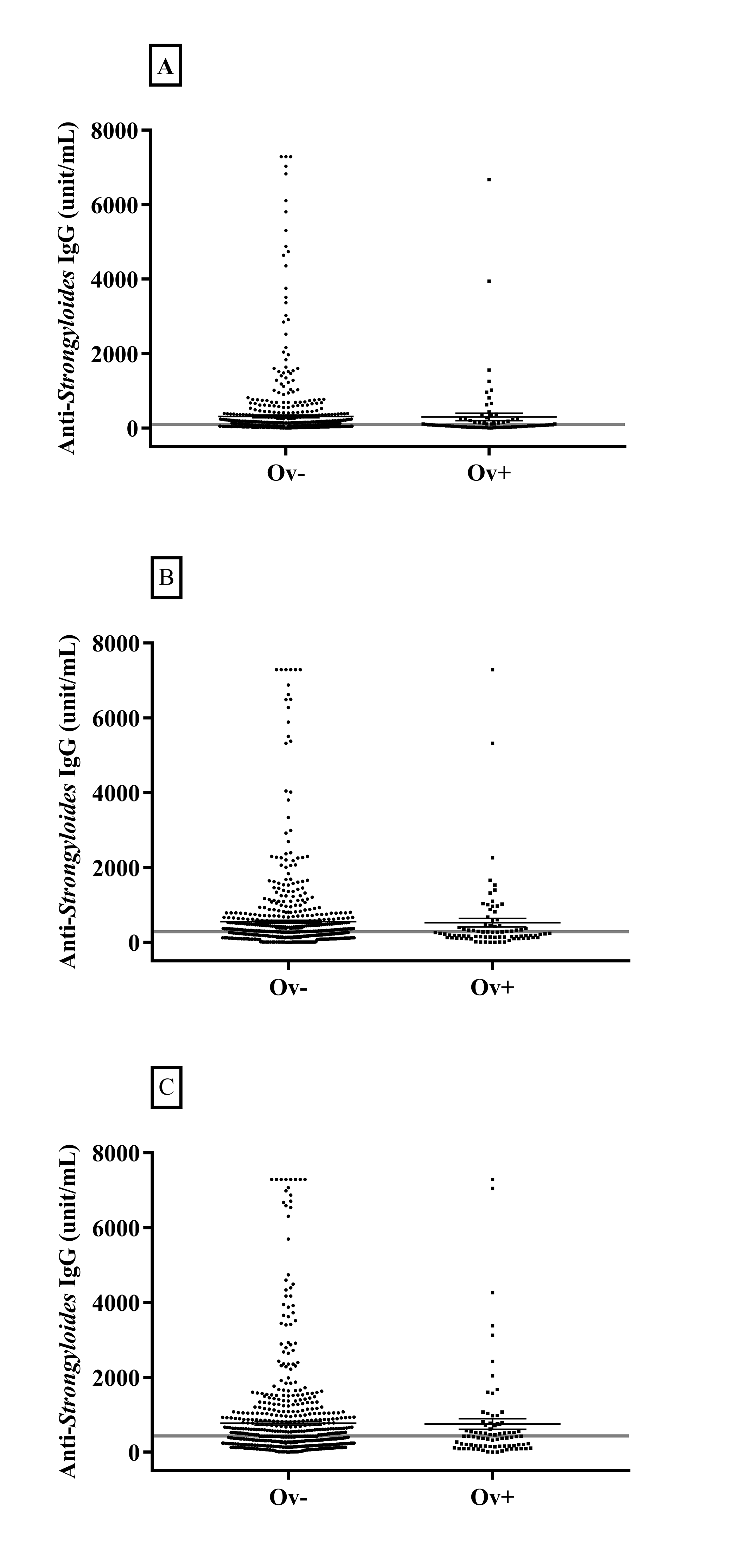

Supplement: S1 Fig — Distribution of anti-Strongyloides IgG by Sr-ELISA (A), Ss-ELISA (B), and NIE-ELISA (C) among individuals with and without parasitologically confirmed opisthorchiasis. The gray horizontal lines represent the cutoff values. Short black lines indicate the mean with lower and upper standard error. Ov- means negative O. viverrini egg; Ov+ means positive O. viverrini egg. (TIF) [file pone.0284305.s001.tif]
