# Supplementary material for: Epidemiology of strongyloidiasis determined by parasite-specific IgG detections by enzyme-linked immunosorbent assay on urine samples using Strongyloides stercoralis, S. ratti and recombinant protein (NIE) as antigens in Northeast Thailand
Source: PLoS One. 2023 Apr 12;18(4):e0284305. doi: 10.1371/journal.pone.0284305 (PMC10096234; doi:10.1371/journal.pone.0284305)
Supplement: S1 Table — Data shown are correlation coefficients between antibody levels (IgG antibody unit/mL) from each pair of ELISA protocols according to Spearman correlation tests and p values (based on data from 966 individuals). (DOCX) [file pone.0284305.s002.docx]

**S1 Table.** Correlations between levels of parasite-specific IgG in urine determined by ELISA protocols using antigen prepared from *S. ratti* (Sr-ELISA), *S. stercoralis* (Ss-ELISA) and recombinant protein NIE antigen (NIE-ELISA). Data shown are correlation coefficients between antibody levels (IgG antibody unit/mL) from each pair of ELISA protocols according to Spearman correlation tests and p values (based on data from 966 individuals).

| Diagnostic methods | Correlation coefficients based on cumulative three-day data | p value |
| --- | --- | --- |
| Sr-ELISA & Ss-ELISA | 0.863-0.603 | < 0.0001 |
| Sr-ELISA & NIE-ELISA | 0.658-0.465 | < 0.0001 |
| Ss-ELISA & NIE-ELISA | 0.724-0.446 | < 0.0001 |
